# Assessment of cognitive functions in patients with multiple sclerosis applying the normative values of the Rao’s brief repeatable battery in the Portuguese population

**DOI:** 10.1186/s12883-021-02193-w

**Published:** 2021-04-21

**Authors:** Claudia Sousa, Mariana Rigueiro-Neves, Ana Margarida Passos, Aristides Ferreira, Maria José Sá, on behalf of the Group for Validation of the BRBN-T in the Portuguese MS Population, Rui Pedrosa, José Vale, Paulo Alegria, Lívia Sousa, Sandra Perdigão, José Leal Loureiro, Andreia Sá, Fanny Lima, Marta Santos, Telma Miranda

**Affiliations:** 1 MS Clinic, Department of Neurology, Centro Hospitalar Universitário São João Porto, Alameda Prof. Hernâni Monteiro, Porto, Portugal; 2 Neuropsychological Unit, Department of Psychology, Centro Hospitalar Universitário São João Porto, Alameda Prof. Hernâni Monteiro, 4200 – 319 Porto, Portugal; 3 BRU-IUL, Instituto Universitário de Lisboa (ISCTE-IUL), Lisbon, Portugal; 4 Faculty of Health Sciences, University Fernando Pessoa, Porto, Portugal

**Keywords:** Multiple sclerosis, Cognition, Neuropsychological test, Brief repeatable battery of neuropsychological tests, Normative data, Cognitive assessment

## Abstract

**Background:**

The Brief Repeatable Battery of Neuropsychological Tests (BRBN-T) is one of the most sensitive and used measures for detecting cognitive impairment in Multiple Sclerosis (MS).

**Objective:**

The aim of this study was to adapt and validate this battery to the Portuguese population of MS patients.

**Methods:**

The Portuguese version of the BRBN-T was applied to a stratified control national sample of 326 individuals considering sex, age, educational level and geographic location and also a clinical sample of 115 MS patients from several national hospitals. Through the exploration of its psychometrics properties, the Portuguese BRBN-T norms were produced.

**Results:**

The normative data is presented as a regression-based formula to adjust test scores for gender, education and age, and the results reveal the BRBN-T has the ability to differentiate between MS patients and healthy participant’s cognitive performance.

**Conclusion:**

This study demonstrated in our clinical population a good ability to detect cognitive impairment. Its clearly contributed to reinforcing the neuropsychological assessment in Portugal in patients with MS, by providing a new set of instruments, which can be used in the clinical practice, and in future studies. Moreover, it will allow a rigorous and precise support in relation to neuropsychological assessment for future longitudinal studies and clinical trials.

## Background

Multiple Sclerosis (MS) is a chronic inflammatory and demyelinating disorder of the central nervous system that can lead to a variety of clinical manifestations, including to a decline of cognitive abilities [1]. Yet, cognitive symptoms may go unnoticed by patients and remain undetected when performing the routine neurological evaluation, unless specific assessment tools are applied.

Cognitive impairment has been reported to affect between 34 to 65% of MS patients [1–3], irrespective of age and gender [4, 5]. It can occur at all stages of the disease since its onset [6, 7], and it tends to progress over time, creating a significant impact on patients´ functioning and quality of life [3, 8–11].

In 1990 the Cognitive Function Study Group of the American National MS Society published the Brief Repeatable Battery (BRB) [12], later simplified as the Brief Repeatable Battery of Neuropsychological Tests (BRBN-T) [1], and through this screening tool a characteristic pattern of cognitive impairment in MS was defined: memory, information processing efficiency, executive functioning, attention and processing speed being the most commonly compromised functions [13, 14]. Other neuropsychological evaluation tests were later designed for MS patients, as the Minimal Assessment Cognitive Function in Multiple sclerosis (MACFIMS) [15, 16], and most recently the Brief International Cognitive assessment Multiple Sclerosis (BICAMS) [17], the latter having been recently validated for use in the Portuguese population by our research group [18].

The BRBN-T, the most widely used battery in MS, is made up of five tests that were found to be the most sensitive for detecting cognitive impairment in MS: the Selective Reminding Test (SRT), the 10/36 Spatial Recall Test (SPART), the Symbol Digit Modalities Test (SDMT), the Paced Auditory Serial Addition Test (PASAT) and the Word List Generation test (WLG) [12]. It is noteworthy that cognitive impairment, as measured by BRBN-T, has been associated with long-term progression of MS [19]. Although the importance of this battery is unquestionable, as testified by its usage in many MS centres and by the inclusion of some of its tests in clinical trials, the unavailability of normative values for specific populations limits a thorough utilization in clinical practice.

In this study we aimed to evaluate the cognitive functions of a representative sample of MS patients in several centers of our country with the BRBN-T and to compare the results with normative values obtained in the Portuguese population that have been partially published, namely the PASAT [20] and SRT tests [21].

The Portuguese language is the 7th most spoken language in the world with more than 242 million native speakers and therefore, this study is of particular importance since it advances the introduction of MS neuropsychological evaluation within Portuguese speaking countries. Furthermore, to our knowledge, this is the first study addressing the neuropsychological evaluation of Portuguese patients with MS and healthy subjects employing the BRBN-T.

## Material and methods

### Participants

The BRBN-T was applied to a group of 115 MS patients (clinical sample) with MS diagnosed according to the McDonald criteria [22], and to a group of 326 healthy control subjects that were matched for age, gender and educational status. (HC sample) (Table 1). Inclusion criteria for both samples were: being aged 20 years or older, having Portuguese as the native language, having attended school in Portugal and having finished primary school.

**Table 1:** Demographic and clinical characteristics (MS – Healthy controls)

	MS patients (*N* = 115)		Healthy controls (*N* = 326)	
	*Mean*	*SD*	*Mean*	*SD*
**Gender (%M - %F)**	(31–69%)		(39–61%)	
**Age (years)**	34.9	8.8	40.3	14.4
**Education (years)**	13.6	3.8	12.3	4.4
**Duration of MS (years)**	2.4	3.8	–	
**EDSS (Mdn)**	1.0		–	
**MS subtypes**	92.2% *RRMS*; 6.1% *CIS;* 0.9% *PPMS*; 0.9% *SPMS*		**–**	

*MS* Multiple sclerosis, *RRMS* Remitting-remission Multiple sclerosis, *CIS* Clinically isolated syndrome, *PPMS* Primary progressive multiple sclerosis, *SPMS* Secondary progressive multiple sclerosis

The MS patients were consecutively recruited at MS Clinics from six hospitals located in three different regions of the country: North - Centro Hospitalar Universitário São João (Porto) and Unidade Local de Saúde do Alto Minho (Viana do Castelo); Centre - Centro Hospitalar Universitário de Coimbra (Coimbra) and Centro Hospitalar de Entre o Douro e Vouga (Feira), South: Hospital de Santo António dos Capuchos (Lisboa) and Hospital Beatriz Ângelo (Loures), whereas the HC group was recruited from the community and among relatives and friends of MS patients. Exclusion criteria were current or past neurological disorder other than MS, presence of major psychiatric illness, history of learning disability, history of serious head trauma, presence of alcohol or drug abuse, relapse and/or corticosteroid use within 4 weeks preceding the neuropsychological assessment. The majority of MS patients had the relapsing-remitting subtype (MS RR 92.2%), the mean disease duration was 2.4 years and the median EDSS (Expanded Disability Status) score [23] was 1.0 (range: 0–6).

The HC sample distribution is representative of the Portuguese population, according to the 2011 Census [24], and regarding geographical, gender and educational variables. Exclusion criteria were also included for this group: history of neurological disorder, history of serious head injury, presence of a major psychiatric illness, history of alcohol or drug abuse, history of learning disability and of regular usage of antidepressants or anxiolytics. This study sample had been included in previous studies of our group that were conducted with the specific aim of validating individual tests in the Portuguese population, such as in the previously mentioned PASAT [20] and SRT tests [21].

All participants volunteered to participate in this study and gave written informed consent in accordance with the Helsinki Declaration. The Portuguese Data Protection Authority and the ethics committee of all hospital centers involved in this study approved the data collection.

### Neuropsychological test procedures

An initial demographic interview was conducted that was based on a common script that included a demographic questionnaire, medical history, drinking and drug habits and present health status. Well-trained clinical psychologists performed the neuropsychological assessment, in a standardized way in a fixed order under the same circumstances (e.g. interview during the day in a quiet room).

The BRBN-T [1] was administered in the following order: Selective Reminding Test (SRT), 10/36 Spatial Recall Test (SPART), Symbol Digit Modalities Test (SDMT), Paced Auditory Serial Addition Test (PASAT), Word List Generation (WLG), Delayed Recall of the SRT, Delayed Recall of SPART. Administration of the test battery lasted from 28 to 35 min.

The *Selective Reminding Test* (SRT) [25] evaluates verbal learning and memory using a learning task in a paradigm of multiple trials. The list includes 12 words, which the examiner reads at a rate of one word per 2 seconds and the participant is instructed to recall all 12 words. In every consecutive trial only the words that are missed in the preceding one are given to the participant. After 15 min, the participant is asked to recall the 12-word list. The test was scored according to the rules of Buschke and Fuld (1974) procedures and allows for the distinction between short-term and long-term memory components, examining also the consistency of retrieval from long-term memory. The SRT indexes used in this study were: Total Recall, Long Term Storage (LTS), Consistent Long Term Retrieval (CLTR) and Intrusions. Total Recall consists of the sum of the recalled items in each trial; LTS is defined as any word that is recalled in two consecutive trials, without the patient being reminded of the word between trials, regardless of him/her forgetting it in subsequent trials; if a word is LTS and is consistently recalled in all subsequent trials (but not just the last trial), then it is scored as an CLTR whereas intrusions of extra-list words are also recorded.

The *10/36 Spatial Recall Test* (SPART) [12] assesses visual memory acquisition and delayed recall. It consists of a 6 × 6 checkerboard with 10 checkers randomly placed. The board is presented to the participant for 10 s. After the presentation, he/she attempts to reproduce the original design on an empty board. This process is repeated twice and after 15 min and in a delay recall trial, the subject is asked to recall the design again. The score is the total number of correct responses for the three trials and the delayed recall trial.

The *Symbol Digit Modalities Test* (SDMT) [26] examines sustained attention, concentration and processing speed. The participant examines a series of nine meaningless geometric symbols, which are labeled from 1 to 9. Then, during 90 s the participants are instructed to replace the symbols in a row by the corresponding number in a verbal manner. The score is the number of correct substitutions (SDMT).

The *Paced Auditory Serial Addition Test* (PASAT) [12] measures sustained attention and information processing speed. In the trial set, a standard series of 61 numbers from 1 to 9 is presented and single digits are shown every 3 s. The participant is instructed to add the number most recently presented to the one immediately preceding it. Thus, the second is added to the first, the third to the second, the fourth to the third, and so on. For example, given the series of numbers 1, 2, 3, 4, the participant must add and provide a verbal response to the digit pairs “1, 2”, “2, 3”, “3, 4”. The PASAT performance is scored manually upon test completion by listening to the participants’ responses and coding the correct responses. The participant is able to complete this test if he provided at least two correct answers in any of the three practice sequences. The test score is the number of correct answers.

The *Word List Generation* (WLG) [12, 27] is a phonemic verbal fluency test that evaluates the spontaneous production of words and mental flexibility, when given a letter from the alphabet and within a limited amount of time (1 minute). The scoring procedures consist of totaling the correct responses.

### Statistical analyses

We followed the statistical procedure suggested by Diehr and colleagues (2003) [28] and Amato and colleagues (2006) [2] to generate the BRBN-T Portuguese norms. Mean values for each BRBN-T test were compared between the MS patients and the healthy controls with the Student’s t-test for independent samples. We also used t-test or one-way ANOVA to test differences between groups regarding their performance on the BRB based on demographic characteristics (gender, age, and educational level).

We analyzed the distribution of the row scores of each BRBN-T test in the two different samples using the Shapiro-Wilk test. For non-normal distributions, we applied an inversed logarithmic transformation before calculating the Z scores [20]. We then transformed these distributions into normally distributed scale scores (Mean = 10; SD = 3).

Stepwise multiple regression analysis was used to screen for the demographic variables (gender, age and educational level) that significantly contribute to predict scale scores in each BRBN-T test in the two different samples. To generate the T-scores corrected for significant demographic variables we performed another multiple regression analysis with these significant variables and saved the unstandardized predicted values for each equation. These values were then rescaled to a T-score (mean = 50; SD = 10) [20, 28].

All statistical analyses were performed using the IBM SPSS version 23.0.

## Results

The results of the BRBN-T neuropsychological tests are presented in Table 2. The comparison of MS patients with healthy control participants showed that groups significantly differed on PASAT and WLG. We also investigated whether there were significant differences between groups regarding their performance on BRBN-T tests.

**Table 2:** BRBN-T mean raw scores (and standard deviations) for multiple sclerosis patients (MS) (*N* = 115) and healthy controls participants (HC) (*N* = 326) per gender, age, level of education and the total sample

	PASAT		SRT - LTS		SRT - CLTR		SPART		SPART -D		SDMT		WLG	
	MS	HC	MS	HC	MS	HC	MS	HC	MS	HC	MS	HC	MS	HC
Gender														
Female	42.92 (11.61)	45.19 (10.53)	49.15 (12.63)	45.72 (14.33)	41.01 (15.59)	37.23 (15.63)	21.72 (5.23)	21.04 (5.00)	7.75 (2.09)	7.53 (2.20)	55.77 (10.98)	55.92 (13.55)	32.84 (7.77)	36.93 (10.62)
Male	44.19 (9.82)	48.88 (9.23)	38.33 (15.04)	40.49 (15.29)	27.31 (15.15)	32.47 (15.00)	19.83 (5.34)	22.38 (4.79)	6.72 (2.46)	8.10 (1.91)	48.56 (12.09)	54.61 (11.25)	30.03 (8.50)	33.35 (9.22)
Age														
20–30	42.76 (12.08)	46.26 (9.12)	46.55 (14.20)	49.47 (13.00)	38.61 (14.81)	41.51 (14.73)	22.92 (5.27)	23.32 (4.17)	8.32 (2.26)	8.51 (1.64)	58.11 (9.35)	63.60 (9.25)	30.55 (7.92)	36.99 (9.75)
31–40	43.96 (11.21)	48.92 (9.90)	46.43 (13.98)	43.42 (14.54)	38.19 (15.57)	35.52 (14.46)	20.23 (5.30)	23.66 (4.14)	7.11 (1.89)	8.52 (1.70)	53.06 (11.05)	59.03 (9.46)	33.38 (7.76)	36.48 (9.48)
41–50	43.64 (9.55)	47.48 (9.47)	45.60 (13.57)	43.85 (13.66)	34.44 (15.60)	35.50 (14.10)	20.92 (5.02)	20.48 (4.86)	7.04 (2.48)	7.29 (2.26)	50.08 (13.29)	53.50 (8.98)	31.08 (8.65)	37.56 (9.73)
51–60	40.00 (10.98)	44.51 (11.65)	34.40 (20.55)	40.61 (14.14)	20.00 (14.41)	31.74 (13.73)	17.00 (3.54)	18.49 (5.05)	5.60 (2.30)	6.58 (2.27)	40.00 (13.19)	46.96 (11.87)	33.60 (9.29)	33.39 (11.33)
61–70		45.25 (12.19)		30.00 (15.66)		20.81 (15.29)		19.19 (5.07)		6.72 (2.07)		39.78 (11.96)		28.41 (8.95)
Education														
Elementary School (4–6)	36.00 (14.01)	42.95 (11.31)	27.82 (15.57)	34.30 (13.68)	19.00 (14.22)	25.20 (14.32)	16.64 (4.52)	18.37 (4.94)	5.91 (1.97)	6.33 (2.37)	36.91 (12.69)	40.67 (11.01)	26.73 (6.80)	28.10 (10.09)
Middle School (7–9)	42.40 (8.19)	43.83 (10.68)	42.70 (12.50)	40.36 (13.24)	31.20 (12.31)	30.96 (11.95)	21.10 (4.99)	20.45 (5.26)	6.50 (2.88)	7.38 (2.19)	49.10 (11.70)	51.94 (9.87)	27.70 (7.68)	33.03 (7.17)
High School (10–12)	43.11 (10.80)	47.67 (9.32)	46.32 (14.59)	44.79 (15.02)	37.75 (15.58)	36.89 (14.86)	22.16 (4.95)	22.67 (4.57)	7.98 (1.77)	8.12 (1.92)	54.68 (9.15)	59.95 (10.09)	30.86 (6.33)	35.31 (8.60)
University (> 12)	45.28 (10.66)	49.69 (8.81)	49.84 (10.86)	50.37 (13.12)	40.82 (13.73)	35.38 (15.54)	21.22 (5.47)	23.16 (4.01)	7.46 (2.39)	8.50 (1.55)	57.02 (10.56)	62.18 (9.20)	34.92 (8.74)	41.75 (9.86)
Total Sample	43.32 (11.06)	46.63 (10.19)	45.77 (14.28)	43.68 (14.91)	36.72 (15.59)	35.38 (15.54)	21.13 (5.31)	21.56 (4.96)	7.43 (2.24)	7.75 (2.11)	53.51 (11.76)	55.41 (12.70)	31.96 (8.08)	35.53 (10.23)
	*t*_(439)_ = 2.92; *p* = .004		*t*_(439)_ = −1.30; *p* = .194		*t*_(439)_ = −.80; *p* = .426		*t*_(439)_ = .79; *p* = .432		*t*_(439)_ = 1.40; *p* = .163		*t*_(439)_ = 1.40; *p* = .161		*t*_(439)_ = 3.80; *p* = .000	

We tested the normality of the distributions and only the distribution of PASAT was not normal in both samples (MS Sample: Shapiro-Wilk = .957; df = 115; *p* < .001; HC Sample: Shapiro-Wilk = .928; df = 326; *p* < .001). Because the distribution of the raw scores was negatively skewed, we applied an inversed logarithmic transformation to reduce these skews using the formula: -log10*(61 – raw scores). We then transformed BRBN-T raw scores tests to scale scores for both samples. Results are presented on Table 3 for the MS patients’ sample and on Table 4 for healthy controls participants.

**Table 3:** Conversion table of raw scores on the BRBN-T to scale scores for the MS Patients (*N* = 115)

PASAT	SRT - LTS	SRT - CLTR	SPART	SPART - D	SDMT	WLG	Scale Score
					0–18		**1**
					–		**2**
			0–10	0–2	19–29	0–14	**3**
	0–19	0–10	11–12	3–3	30–31	15–17	**4**
0–21	20–24	11–14	13–13	–	32–35	18–19	**5**
22–25	25–29	15–18	14–14	4–4	36–39	20–22	**6**
26–33	30–33	19–23	15–16	5–5	40–43	23–25	**7**
34–40	34–38	24–28	17–18	6–6	44–47	26–27	**8**
41–45	39–43	29–34	19–20	–	48–51	28–30	**9**
46–49	44–48	35–39	21–22	7–7	52–55	31–33	**10**
50–52	49–52	40–44	23–23	8–8	56–59	34–35	**11**
53–54	53–57	45–49	24–25	9–9	60–63	36–38	**12**
55–55	58–62	50–54	26–27	10–10	64–67	39–41	**13**
56–57	63–67	55–62	28–29		68–72	42–43	**14**
58–58	68–69	63–65	30–30		73–75	44–48	**15**
–		66–69			–	49–50	**16**
59–59					77–82	51–52	**17**
60–60					83–83	–	**18**
						55–55	**19**
							**20**

**Table 4:** Conversion table of raw scores on the BRBN-T to scale scores for the Heathy Controls Participants (*N* = 326)

PASAT	SRT - LTS	SRT - CLTR	SPART	SPART - D	SDMT	WLG	Scale Score
			0–5	0–1			**1**
	0–8		6–9	2–2	0–23	0–9	**2**
	9–13	0–3	10–10	–	24–27	10–13	**3**
0–17	14–17	4–7	11–12	3–3	28–32	14–16	**4**
18–20	18–22	8–12	13–14	4–4	33–36	17–20	**5**
21–31	23–27	13–17	15–15	–	37–40	21–23	**6**
32–39	28–31	18–22	16–17	5–5	41–44	24–26	**7**
40–44	32–36	23–27	18–19	6–6	45–49	27–30	**8**
45–48	37–41	28–32	20–20	7–7	50–53	31–33	**9**
49–51	42–46	33–37	21–22	–	54–57	34–37	**10**
52–54	47–50	38–42	23–24	8–8	58–61	38–40	**11**
55–55	51–55	43–47	25–25	9–9	62–65	41–44	**12**
56–57	56–60	48–52	26–27	10–10	66–70	45–47	**13**
58–58	61–65	53–57	28–28		71–74	48–50	**14**
–	66–69	58–62	29–30		75–78	51–54	**15**
59–59	70–70	63–67			79–82	55–58	**16**
–		68–70			83–87	59–61	**17**
60–60					88–91	62–66	**18**
					92–92	67–67	**19**
							**20**

In Table 5 we present the results of stepwise multiple regression analysis for all BRBN-T. We used gender (0 for male and 1 for female), age (number of years), education (number of years of education) and also the quadratic terms of age and educations.

**Table 5:** Results from stepwise multiple regression analysis to analyze possible predictors of BRBN-T

	MS Patients (N = 115)			Healthy Controls (*N* = 326)		
	Predictors	β	p	Predictors	β	p
PASAT	Education	.21	.206	Gender	−.23	.000
				Age	.14	.019
				Education	.38	.000
SRT - LTS	Gender	.27	.000	Gender	.13	.008
	Education	.35	.002	Education	.31	.000
				Age^2^	−.21	.000
SRT - CLTR	Gender	.34	.000	Education	.37	.000
	Education	.29	.001	Age^2^	−.18	.001
SPART	Age	−.25	.006	Gender	−.17	.001
				Education	.29	.000
				Age^2^	−.24	.000
SPART - D	Age	−.32	.001	Gender	−.17	.001
				Age	−.28	.000
				Education	.30	.000
SDMT	Education	.42	.000	Age	−.39	.000
	Age^2^	−.29	.000	Education	.46	.000
	Education^2^	−.10	.043	Education^2^	−.69	.000
WLG	Education^2^	.37	.000	Gender	.11	.025
				Education	.46	.000

Based on the results of the stepwise multiple regression analysis we performed additional multiple regression analysis with BRBN-T (scale scores as dependent variable and significant demographics variables in each neuropsychological test as predictors) to generate corrected T-scores. Normative formulas for the Portuguese population and MS patients are presented in Table 6.

**Table 6:** BRBN-T normative formulas to calculate T-scores for the Healthy Controls and MS Patients

	Multiple Sclerosis Patients	Healthy Controls
PASAT	PASAT T score = 22.818 + (3.393*Scale score) + (− 0.495*Education)	PASAT T score = 28.875 + (3.626*Scale score) + (− 0.157*Age) + (5.296*Gender) + (− 0.981*Education)
SRT - LTS	LTS T-Score = 30.652 + (3.827*Scale) + (− 6.674*Gender) + (− 1.052*Education)	LTS T-Score = − 8.665 + (7.448*Scale) + (0.004*Age^2^) + (− 5.198*Gender) + (− 1.501*Education)
SRT - CLTR	CLTR T-Score = 29.317 + (3.843*Scale score) + (− 8.453*Gender) + (− 0.876 *Education)	CLTR T-Score = − 6.733 + (7.371*Scale score) + (0.003*Age2) + (− 1.777 *Education)
SPART	SPART T-Score = 5.204 + (3.446*Scale score) + (0.297*Age)	SPAR T-Score = 14.988 + (3.773*Scale score) + (0.002*Age2) + (3.894*Gender) + (− 0.739 *Education)
SPART - D	SPART-D T-Score = 1.795 + (3.512*Scale score) + (0.375*Age)	SPART-D T-Score = 11.951 + (3.773*Scale score) + (0.181*Age) + (3.914*Gender) + (− 0.764 *Education)
SDMT	SDMT T score = 36.669 + (4.072*Scale score) + (0.005*Age^2^) + (− 4.032 *Education) + (0.106*Education^2^)	SDMT T score = 16.655 + (4.955*Scale) + (0.379*Age) + (− 3.914*Education) + (0.098*Education^2^)
WLG	WLG T score = 22.168 + (3.580*Scale score) + (− 0.040 *Education^2^)	WLG T score = 28.116 + (3.828*Scale score) + (− 2.589*Gender) + (− 1.207 *Education)

We adopted the same statistical procedure from a previous study [29] in order to determine the performance of each BRBN-T. Performance below the 5th percentile of the normative sample on at least two out of five tests of the BRBN-T was considered as criteria for cognitive impairment. Then, using the previously reported criteria of impairment defined by “two or more abnormal tests” [12], it was found that 30.43% of the MS sample was impaired at baseline.

In the current study we calculated the following scores: accuracy, sensitivity, specificity, area under the ROC curve (AUC), positive likelihood ratio (PLR), negative likelihood ratio NLR, positive predictive value (PPV) and negative predictive value (NPV). All the scores were calculated through the MedCalc statistical software. Accuracy represents the proportion of true positive scores in the studied population, considering both the true positive and the true negative values (Number of correct / positive assessments / Number of all assessments). Sensitivity is the ratio between the number of true positives and the number of total cognitive impaired patients (Number of true positive assessment / Number of all positive assessments). Specificity is the ratio between the number of true negatives and the number cognitive unimpaired patients (Number of true negatives assessment / Number of all negative assessments). The AUC allowed us to evaluate the accuracy of the diagnostic test. Large ROC areas represent more accurate diagnostic tests with values of 0.9 < AUC < 1.0 considered as excellent, 0.8 < AUC < 0.9 very good, 0.7 < AUC < 0.8 good, 0.6 < AUC < 0.7 sufficient, 0.5 < AUC < 0.6 bad, and AUC < 0.5 as not useful. Likelihood ratio is an important measure for ruling-in/out diagnosis. The positive likelihood ratio (PLR) explains how much more likely the positive test is to occur in patients with cognitively impaired from cognitively unimpaired patients with MS. The higher the test score, the more indicative it is of MS with values higher than 10 meaning that the test has a significant contribution to the diagnosis. Negative likelihood ratio (NLR) explains how much less likely is the negative test score to occur in a MS patient than in a healthy participant. Good diagnostic tests have scores lower than 0.1. Finally, PPV represents the ratio between the number of true positives and the number of positive tests, while NPV represents the ratio between the number of true negatives and the number of negative tests.

Results in Table 7 represent the performance of each of the seven BRBN-T tests, considering a sample of 110 patients with MS. Accordingly, there is evidence that SDMT and SRT are the most sensitive tests. Taking into consideration the area under the ROC curve (AUC), SRT has a classification of good (AUC = 0.74) and SDMT of very good (AUC = 0.82). Accordingly, these results support the accuracy of both tests, suggesting their discriminative power to distinguish cognitively impaired from cognitively unimpaired MS patients. If we take into account the positive likelihood ratio, four tests (SRT, RLP, SDMT, and RCLP) have a higher score than 10. Thus, results provide evidence that, for these tests (as expected), it is more likely that the positive test will occur in cognitively impaired than in cognitively unimpaired patients with MS. However, our findings concerning the Accuracy of single test parameters should not be overestimated. Findings from previous research regarding the BRBN-T state that the differentiation of cognitively impaired from cognitively unimpaired patients with MS is not feasible with one screening test alone (such as the SDMT) [30, 31]. Therefore, we advise that cognitive impairment in patients with MS should be assessed with BICAMS or a short version of the BRB to provide more accurate results.

**Table 7:** Performance of each of the studied tests for the MS sample

	Accuracy	Sensitivity (95% CI)	Specificity (95% CI)	AUC (95% CI)	PLR (95% CI)	NLR (95% CI)	PPV (95% CI)	NPV (95% CI)
RLP	83%	18% (5–40)	99% (94–100)	.59 (.49–.68)	16 (1.88–136)	.8 (.7–1)	80% (32–97)	83% (80–86)
RCLP	82%	14% (3–35)	99% (94–100)	.56 (.46–.66)	12 (1.31–110)	.9 (.7–1)	75% (25–96)	82% (79–84)
SRT	88%	50% (28–72)	98% (92–100)	.74 (.65–.82)	22 (5–92)	.5 (.3–.8)	85% (57–96)	89% (84–92)
SPART	83%	36% (17–59)	94% (87–98)	.65 (.56–.74)	6 (2–18)	.7 (.5–.9)	62% (37–82)	86% (81–89)
SDMT	90%	68% (45–86)	95% (89–98)	.82 (.73–.89)	15 (6–41)	.3 (.2–.6)	79% (58–91)	92% (87–96)
PASAT	84%	41% (21–64)	94% (87–98)	.68 (.58–.76)	7 (3–19)	.6 (.4–.9)	64% (40–83)	86% (82–90)
WLG	79%	32% (14–55)	91% (83–96)	.61 (.52–.70)	4 (1–9)	.8 (.6–1)	47% (26–68)	84 (80–88)

## Discussion

In this multicentre study, we evaluated the cognitive functioning of Portuguese patients with MS and healthy controls with the BRBN-T battery. Both samples were collected in different regions of the country, and should therefore be representative of the entire population. This is the first study carried out in Portugal with the aim of validating the BRBN-T battery and its applicability in patients with MS, which is a relevant task since the prevalence of the disease in Portugal has recently been estimated at around 64.4 per 100,000 inhabitants [32].

Our study provides both normative data for the A version of the BRBN-T in a Portuguese population and also the gross scores corrected for the relevant demographic factors. This study reinforces the importance of using demographically corrected scores for discriminating between persons with and without cognitive impairment in MS. Considering the great contribution of demographic characteristics to cognitive performance, the literature suggests that regression-based norms do not categorize continuous variables, which tend to increase specificity and efficiency of estimation, allowing the adoption of reduced sample sizes [33, 34].

In order to better comprehend the performance of patients with MS in each BRBN-T test, we compared that performance with the results obtained in a normative sample covering a larger number of subjects, which was an important advantage, as normative studies conducted in other countries included smaller samples and only healthy participants [35, 36]. Boringa and colleagues were the first to investigate and document the impact of demographic factors, such as age, sex and education on the cognitive performance of BRBN-T in healthy participants [35]. In our sample, the mean age (40.3 ± 14) was similar to that reported in the Italian (41.5 ± 9.8) and Dutch (45.8) groups [34, 36]. It is known that age is one of the main factors that influence test scores. In our study and in consistence with others [35, 36], increasing age was associated with poorer performance across the entire battery, except for the verbal fluency test, the WLG. The impact of age on MS patients has also been observed in SPART and SDMT [37], revealing that older patients presented with increased changes in the capacity of visuo-spatial memory and the speed of information processing. On the other hand, only few studies included young patients with MS and reveal that in early-onset MS population mostly failed in spatial memory and semantic fluency [38].

Regarding the educational level, we found that compared to other studies [39, 40] we obtained a similar higher average educational level with low standard deviation (12 + 4 vs. 12.3 + 3.6). We found in both groups a strong relationship between these variables, showing that the higher the individual’s level of education, the better the cognitive performance. This relationship was observed in the clinical group, with the exception of the SPART test. Better performance in PASAT 3.0 was observed in our MS patients with a higher level of education like in another research [37], perhaps because these patients are active professionally and are more likely to be more cognitively trained.

The gender ratio in normal individuals and patients with MS was matched. In a previous study [40], no gender differences were found, except for WLG, in which women were better than men. In the present study, we found gender to have an impact in the control group in all tests with the exception of CRLT and SDMT. In the clinical sample, differences between genders were found only in the selective memory test, with women performing better than men.

The analysis of our results in the MS population benefited from previous normative data obtained for the PASAT [20] and SRT [21] tests. The results show that SDMT and SRT are the most sensitive tests with the greatest discriminative power. This evidence is in line with previous studies [29] that showed that both tests are very sensitive for detecting cognitive impairment in patients with multiple sclerosis. In terms of validity, both tests measure the cognitive dimensions of verbal memory, concentration and sustained attention, which represent cognitive impairments in patients with MS [13].

BRBN-T tasks were able to identify cognitive impairment in 30.43% of patients with MS using the impairment criteria defined by two or more abnormal tests. Similar values were reported in other studies, ranging from 34 to 65% [1–3, 31]. Our results showed that 46.42% of patients not yet treated with disease-modifying drugs had cognitive impairment, which is in line with the findings of other studies which showed that cognitive symptoms are already present in the early stages of the disease and sometimes even before the onset of clinical symptoms and signs [6, 7]. The assessment of cognitive functioning at the beginning of the disease course has shown to identify individuals with cognitive impairment, as well as to predict future changes, limitations and disease progression. In this sense, the early identification of cognitive changes can lead to more timely and targeted interventions.

This study has some limitations. Firstly, the tests were carried out in a low number of different disease phenotypes; secondly, there was a low level of physical disability with a mean EDSS of 1.0 in the MS group and lastly, the study did not include early-onset MS patients and in both samples there was a low number of patients above 60 years of age.

These data should be extrapolated with cautions for other MS phenotypes, for patients with higher EDSS and early-onset patients and over 60 years of age because these groups are underrepresented in this study leading to bias in the determination of cognitive impairment.

## Conclusions

Cognitive impairment has been increasingly investigated in MS patients, not only in the clinical setting as a means to reach a thorough and comprehensive understanding and management of the disease, but also as an important outcome in clinical trials. Early detection of cognitive impairment is particularly important in MS as it is a chronic and progressive neurological disease that usually begins in young age. Besides early treatment with disease-modifying drugs, MS patients with cognitive deficits certainly benefit from other therapeutic strategies, rehabilitation, compensatory measures and lifestyle adjustments to manage those disabling symptoms. To match that purpose, an essential prior stage of the rehabilitation process is the evaluation of cognitive functions using validated instruments in a specific population, as we have carried out in Portugal and herein describe. In addition, the present study enables the comparison with the results obtained in MS populations from other countries.

## Data Availability

The datasets used and analysed during the current study are available from the corresponding author on reasonable request.

## Abbreviations

MS: Multiple sclerosis
RRMS: Remitting-remission Multiple sclerosis
CIS: Clinically isolated syndrome
PPMS: Primary progressive multiple sclerosis
SPMS: Secondary progressive multiple sclerosis
BRB: Brief Repeatable Battery
BRBN-T: Brief Repeatable Battery of Neuropsychological tests
BICAMS: Brief International Cognitive Assessment for Multiple Sclerosis
SRT: Selective Reminding Test
SPART: 10/36 Spatial Recall Test
PASAT: Paced Auditory Serial Addition Test
SDMT: Symbol Digit Modalities Test
WLG: Word List Generation test
MACFIMS: Minimal Assessment of Cognitive Function in Multiple Sclerosis
HC: Healthy subjects
EDSS: Expanded Disability Status Scale
IBM SPSS: Statistical Package for the Social Sciences
